# Distal tibial tubercle osteotomy: A descriptive analysis of factors that influence bone healing at the distal bony interface

**DOI:** 10.1002/jeo2.70872

**Published:** 2026-07-30

**Authors:** Arthur Only, Marta Engelking, Julie Agel, Elizabeth Arendt

**Affiliations:** ^1^ Department of Orthopedic Surgery University of Minnesota Minneapolis Minnesota USA; ^2^ St. Croix Health Croix Falls Wisconsin USA

**Keywords:** bony healing, distalization, gap resorptiontibial tubercle

## Abstract

**Purpose:**

To evaluate factors that could impact bone healing following a tibial tubercle osteotomy distalization (dTTO) and the healing of the osteotomized tibial bone (OTB), focusing on its distal extent, where it contacts the anterior tibial shaft (ATS). Secondary aims: to document the average healing time at the dTTO interface, to evaluate frequency of postoperative bone absorption at the distal osteotomy interface, and whether the gap is related to complications of tibia fractures/delayed unions.

**Methods:**

Consecutive distal TTO patients were retrospectively reviewed including demographic data and distal gap measurements on intraoperative/postoperative sagittal knee radiographs. ‘Gap resorption’ was defined as >2 mm increase in the gap between intraoperative and follow‐up radiographs.

**Results:**

A total of 101 knees underwent dTTO (2009–2015), 73 females/28 males; mean age(range): age 21 years old (13–45). 88% had x‐rays that allowed assessment within 4 months. 62% had radiographic healing at the distal OTB‐ATS. The initial gap was (mean [range]): 1.48 mm (0–8.4). All but two patients had initial gaps <3.3 mm. Eleven per cent had an increase in their distal bony gap >2 mm (range 2–7.8 mm) without radiographic evidence of motion at point of screw fixation. The mean thickness of the OTB as a % of total tibial width was 31% (range 22%–37%). There were 4 tibial fractures, none statistically related to initial gap distance, mm of distalization or %thickness of the osteotomy segment. Seven patients had gap resorption >2 mm, 2 with tibia fracture. Fracture time from surgery was 18–201 days.

**Conclusion:**

Of the factors reviewed in this cohort, no factors were identified directly relating to risk of tibia fracture or bone healing except gap resorption >2 mm at OTB‐ATS interface. At 4 months complete radiographic healing was present in 62%. Bone resorption at the distal OTB‐ATS interface is recognised postoperatively (11%). Though not statistically correlated with timely healing, it may be a risk factor for tibial shaft fracture.

**Level of Evidence:**

Level III, retrospective comparative study.

**Abbreviations**
ATS: anterior tibial shaft
BMI: body mass index
CDI: Caton‐Deschamps Index
dTTO: tibial tubercle distalization
ISR: Insall‐Salvati Ratio
LPD: lateral patellar dislocation
MPFL‐R: medial patellofemoral reconstruction
OTB: osteotomiz bone sement
PWB: protected weight bearing
TS‐TT: tibial tubercle‐tibial shaft
TT: tibial tubercle
TTO: tibial tubercle osteotomy

## INTRODUCTION

Lateral patellar dislocation (LPD) is defined as disruption of the normal anatomic tracking alignment of the patella within the patellofemoral joint [15]. Patella alta has been identified as a primary risk factor for LPDs [2, 3] and is defined as an abnormally elevated patella in relationship to the distal femur/trochlear groove or the proximal tibia [5]. The main surgical intervention for correction of patella alta is to distalize the tibial tubercle, first established in the 1980s by Caton‐Deschamps [6]. The repositioning of the tibial tubercle leads to normalising patella height, which initiates earlier patellar engagement in the trochlear groove [12]. Studies have shown significant improvement in pain and function scores in patients with patella alta following distalization tibial tubercle osteotomy (dTTO), making this a beneficial surgical stabilisation procedure for those patients who fail conservative treatment after LPD [10, 11, 16, 17].

However, this is not without drawbacks. In establishing bony fixation, the goal is to secure bone‐to‐bone contact posteriorly and distally. In some cases, a measurable distal gap exists following fixation at time zero, which can be considered a technical issue. At subsequent postoperative radiographic evaluations, there can be additional gap progression, which we have termed ‘gap resorption’. This is defined as an increase in the interface between the distal end of the tibial osteotomy segment and the tibial shaft after intraoperative screw fixation. This increase can be measured without apparent movement noted in the screw fixation on x‐ray. Though gap distance is noted postoperatively, there is little discussion in our literature whether gap distance relates to patient factors or modifiable surgical issues, and whether gap distance is associated with postoperative delayed healing or complications. Assessment of surgical‐related factors associated with bony healing of the osteotomy segment may glean insights into ways to improve outcomes and reduce complications.

The primary aim of this study was to evaluate the technical (surgical) factors and demographic factors that could impact bone healing following a tibial tubercle osteotomy and the healing of the distalized osteotomized tibia bone shingle (osteotmized tibial bone [OTB]); especially at its distal extent where it contacts the cortex of the anterior tibial shaft (ATS). Surgical factors included length of the OTB, width of the OTB as a % of the tibial width, and intraoperative gap. Demographic factors were age, sex, body mass index (BMI) and smoking status. Secondary aims were as follows: to document the average healing time at the tibial tubercle osteotomy interface following distalization, to evaluate the frequency of postoperative bone absorption at the distal osteotomized bone interface (OTB‐ATS) and determine if the width of the gap (both intraoperative and postoperative) at the distal osteotomy interface is related to complications of tibia fractures/delayed unions. It was hypothesised that odds of complications were associated with larger measurable gap sizes at the distal osteotomy interface site.

## METHODS

This study received institutional review board approval.

### Patient selection

This was a consecutive series of a single surgeon's practice, with a high volume of patellofemoral instability patients. This current study is a secondary investigation of an already published data set of patients undergoing medial patellofemoral ligament reconstruction (MPFL‐R) and dTTO for patellar stabilisation [17].

All patients having undergone a dTTO between the years of 2009 to 2015 were identified and selected for study inclusion. Patient exclusion criteria included the following: incomplete radiographic data, absence of follow‐up documenting presence of osteotomy site union or delayed union/nonunion, and patients that underwent tibial tubercle osteotomy without distalization. Patients were also excluded from study inclusion if they required additional osteotomies or concomitant intraarticular procedures.

### Surgical technique

Surgical indications were R‐LPD combined with patella alta. Surgical threshold for d‐TTO was Caton‐Deschamps Index (CDI) or Insall‐Salvati Ratio (ISR) ≥ 1.4, and/or a Patellar trochlear Index < 0.15. All included patients underwent dTTO and MPFL‐R for surgical patellar stabilisation without other concurrent bony procedures and were treated by the same individual surgeon. Postoperative surgical goal for patellar height was CDI = 1.1–1.2. All patients had closed tibial physes.

The length of the bony tibial osteotomy segment was measured directly at the time of surgery by the operating surgeon, and this measurement was recorded in the operative note. The surgical methodology was a distal step cut technique, with thinning of the segment from proximal to distal [1]. Ninety‐seven knees underwent this technique (97%), and four patients had a dovetail technique.

### Rehabilitation

A standardised rehabilitation process was implemented, detailed in an earlier publication [17]. In brief, patients were restricted to protected weight bearing (PWB) with a brace locked in extension for 4 weeks following surgery. The brace was opened when sitting, allowing free range of knee motion. From 4–6 weeks following surgery, patients were continued on PWB with brace unlocked allowing free range of knee motion. Ambulatory aides were discontinued after 6 weeks based on pain‐free ambulation and radiographic healing.

### Outcomes measures

Patient demographic information, clinical exam findings and radiographic measurements were prospectively collected and retrospectively reviewed on consecutive patients with recurrent LPDs. Clinical exam assessments and radiographic measurements were documented at routine postoperative follow‐up appointments. All postop appointments were done at the same institution, and follow‐up radiographs done with the same clinical imaging site and protocol. Specifically, a standing true lateral radiograph of the involved knee was taken at follow‐up [17].

**Figure 1:**
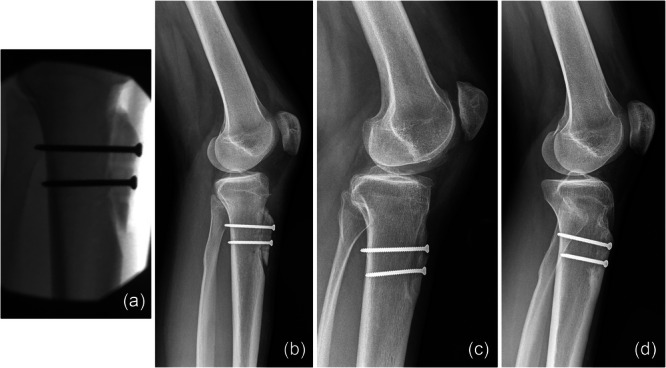
Sagittal tibial image showing (+) intraoperative gap with no gap resorption. Healed without complication. (a) Flouroscopic sagittal image intraop. (b) Sagittal image: 4 week po (Day 38). (c) Sagittal image: 12 weeks postop (d) Sagittal image: 9 months postop.

**Figure 2:**
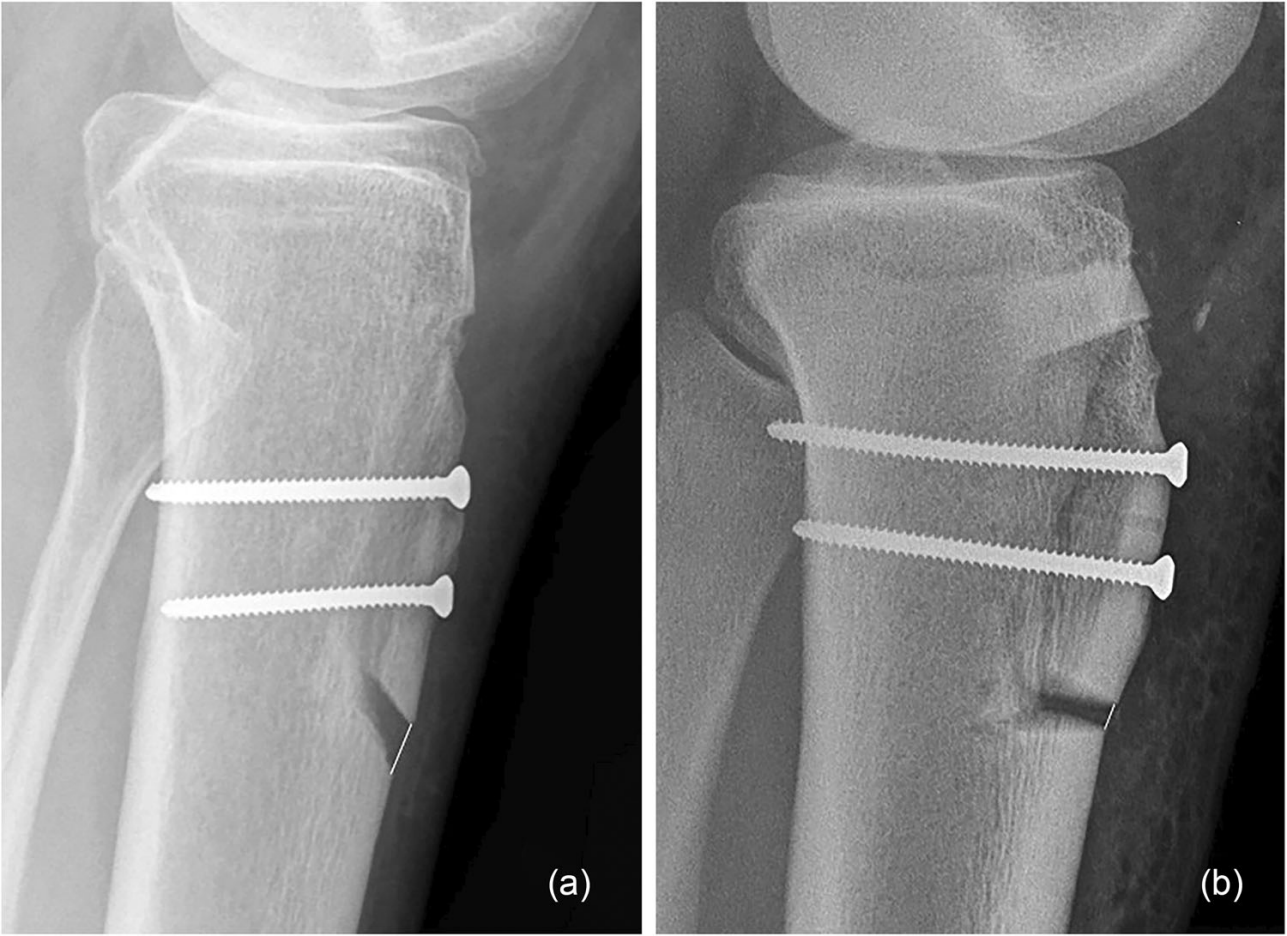
Measuring the gap postop: Sagittal radiograph measuring the gap at the anterior cortical level. (a) Dovetail distal cut with gap measurement 8.4 mm. (b) Step‐cut distal cut with gap measurement 4.1 mm.

**Figure 3:**
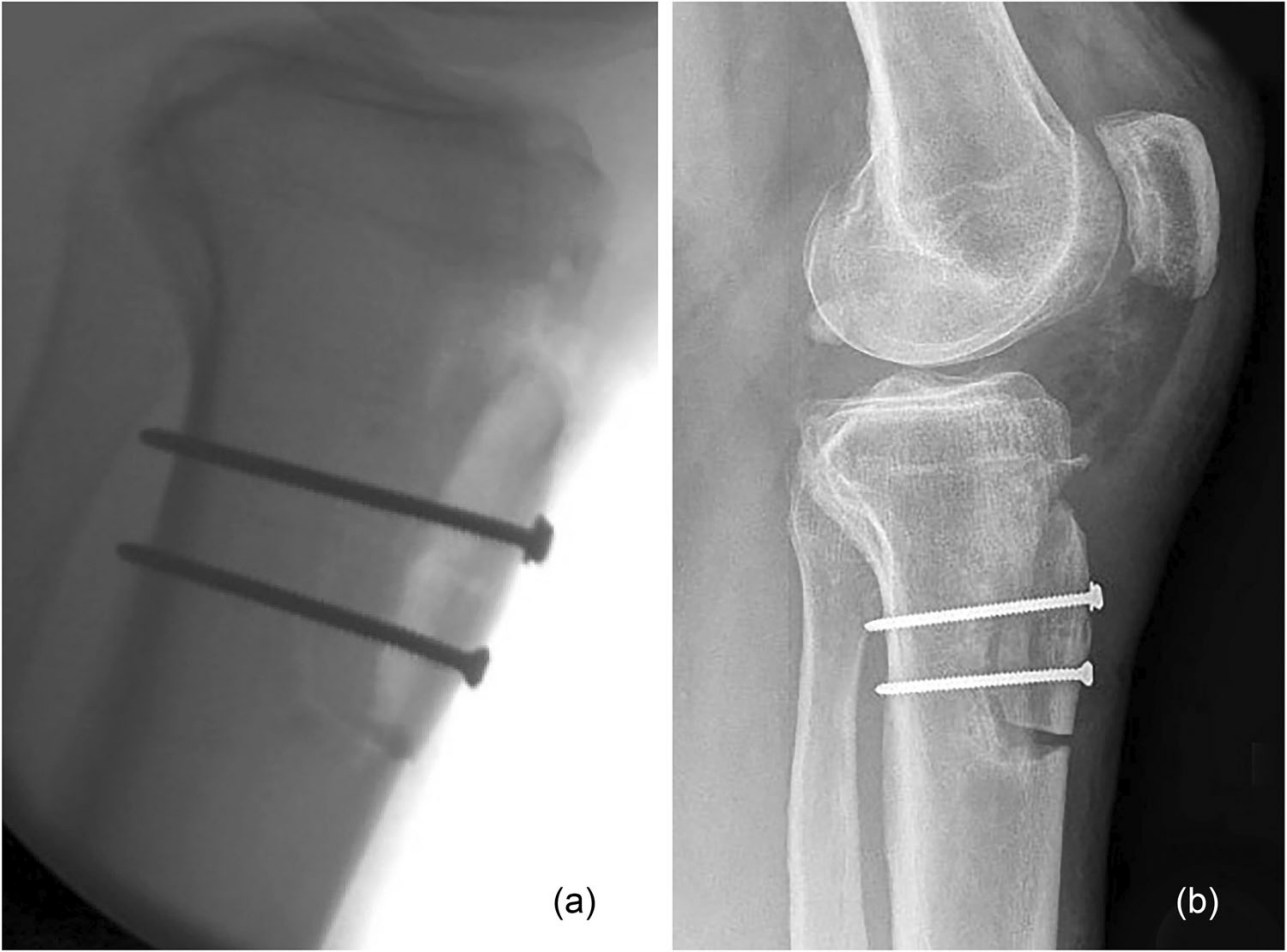
Sagitttal tibial image of post op gap resorption. (a) Flouroscopic image intraop no gap. (b) Sagittal image post op week 7: 2.2 mm gap.

**Figure 4:**
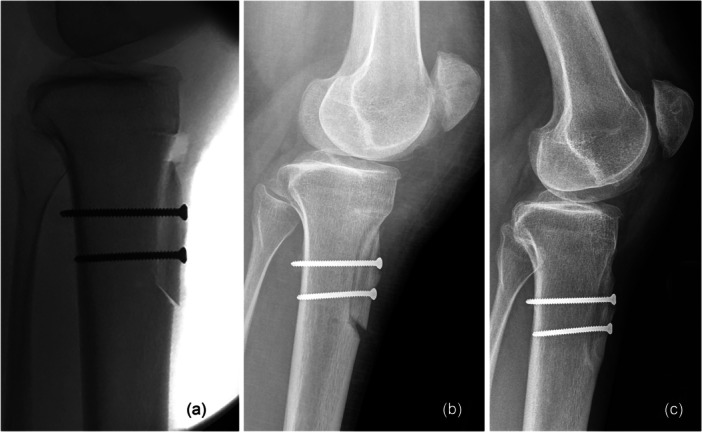
Sagittal image of post op gap resorption. (a) Flouro image intraop. (b) Sagittal image post op Week 4 (33 days): 3.8 mm. (c) Sagittal image post op 6 months postop (healed).

**Figure 5:**
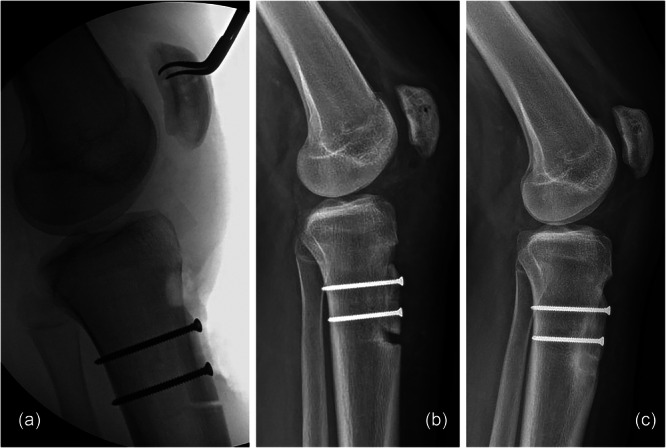
Sagittal image of post op gap resorption. (a) Flouro image intraop. (b) Sagittal image post op Week 5 (38 days): 3.2 mm. (c) Sagittal image post op 6 months postop (healed).

The distal gap was measured intraoperatively (fluoroscopic image) at time zero, and on first and last radiographs at follow‐up visits, until healing was visualised (Figure 1a–d). It was measured at the anterior cortical level (Figure 2a,b). ‘Gap resorption’ was defined as any increase in the gap measurement between the intraoperative measurement and follow‐up measurements (Figure 3a,b) (Figure 4a–c) (Figure 5a–c). A cut‐off of ≥2 mm, empirically derived, was used to represent a meaningful measurable gap.

### Statistical analysis

Descriptive statistics were used for all analyses.

### Data variables

The following demographic information was collected for each patient: age, sex, BMI and smoking status. Smoking cessation was requested by the surgeon preoperatively.

**Figure 6:**
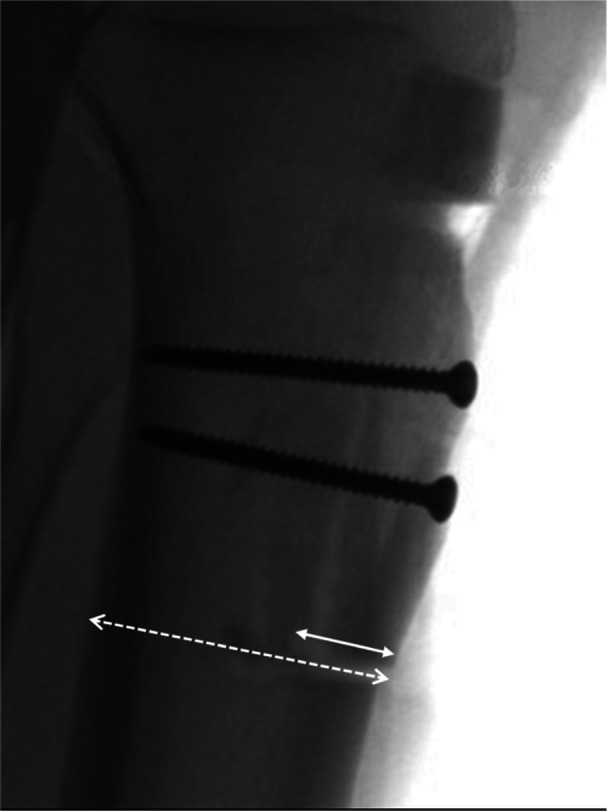
Measuring thickness of the osteotomy segment as a % of the tibial width at the distal end of the tibial segment. Solid line: width of the osteotomy segment at it distal point = 19.8 mm. Dashed line: width of the tibia at that same level = 58.5 mm. Osteotomy width = 34%.

Time to heal was calculated from the date of surgery to first radiograph with complete bony union across the posterior and distal osteotomized segment of tibia bone. The ‘gap’ is defined as the space between distal end of the osteotomized segment and the anterior tibial cortex (OTB‐ATS). Additional measurements of radiographs included the greatest width of the bony block (osteotomized segment) as a percentage of the total tibial width at that level (Figure 6). Intraoperative measurements were made using a calibrated system converting fluoroscopic images measurements to millimetre measurements, based on width of the screw head used in the operation (3 mm). Measurements were taken of the distal bony gap for all follow‐up radiographs obtained until either bony healing or the last date of radiographic follow‐up. All measurements were made by the second author (M.E.), who was blinded to any clinical outcomes. To ensure accuracy, the first four patients were measured by the senior surgeon (E.A.) and these measurements were compared to those obtained by the second author. Outliers, described as the top four largest measurements on radiographs, were also reviewed for accuracy and consistency (intraclass correlation coefficient, 0.983).

## RESULTS

A total of 101 knees (89 patients, 73 female) underwent dTTO were eligible for inclusion. Patients age 13–45 years; mean 21. The mean BMI was 26.2 (17–44). Ten patients were documented tobacco users; two patients had no documentation; the rest identified as nontobacco users.

Intraoperative measurements: Distalization in millimetres (mm) averaged 9.7 mm (range 4–15 mm). The intraoperative gap on fluoroscopic image was mean 1.48 mm (range 0–8.4). Thirty‐two knees had distalization >10 mm; the intraoperative gap on these 32 patients was variable (0–5.4 mm). There was no relationship between millimetres of distalization and gap measurement at any time point postoperatively. The two largest intraoperative gaps were 8.4 mm (with tibial segment distalized 10 mm) and 5.4 mm (with tibial segment distalized 15 mm).

Postoperative gap progression: Eleven knees (11%) had an increase in their distal bony gap >2 mm (gap resorption range 2.0−7.8 mm) without radiographic evidence of motion in the tubercle segment screw fixation. The tibial width (thickness) of the distal end of the osteotomy computed as a percentage of the total tibial width at that same site was a mean of 31% (range 22–37%). [Tibial width mean (range): 48.2 (33.3–68.6), Distal tibial osteotomy width mean (range): 10.1 mm (4.1–18.8)].

Time to heal: Healing is defined as obliteration of the osteotomy site with visible bone across the interface between the distal osteotomy site and the tibia.

Eighty‐nine knees (88%) had x‐rays that allowed assessment within 4 months; 62 knees (70%) had radiographic healing at 4 months. All knees except one had radiographic healing at the 1‐year mark. This one patient had images at 9 months with healing at the OTB‐ATS interface and did not return until the 3‐year mark, at which time the (OTB‐ATS) interface had complete radiographically healing.

Time to heal was not significantly correlated with sex, BMI or smoking status, though the smoking group was too small to make meaningful comparisons. Age showed statistical significance (*p* = 0.02), albeit poor correlation (*r* = 0.29) with days to documented healing. All the younger (<18) subjects healed quicker than the older subjects. Eleven knees had gap resorption >2 mm (range 3.2–7.8), two of which went on to have a tibia fracture (18 days with 3.5 mm gap, 84 days with a 3.2 mm gap). This was not statistically significant.

**Figure 7:**
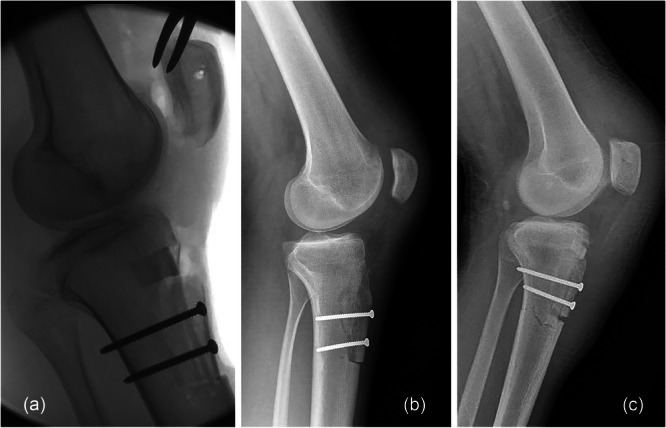
Sagittal tibial image. (a) Interoperative fluoroscopic image. (b) Sagittal radiograph: 18 days postop. (c) Sagittal radiograph 28 days postop with fracture line clearly evident.

There were four total tibial fractures, three of four treated nonoperatively. All fractures emanated from the anterior cortex at the distal ATS location (Figure 7). There were two male patients and two female patients (all nonsmokers). Fracture time from surgery was between 1 and 6 months. (18, 84, 158 and 201 post op days). Gap distances in this fracture group were varied; initial gap ranged between 0.5 and 2.4 mm. Two of four fractures had gap resorption >2 mm (3.2 and 3.5 mm), and both patients fractured in the early postoperative time period. The timeline and mechanism of fracture is detailed in Table 1. Tibial fracture was not statistically related to intraoperative or resorption gap distance at any time point, mm of intraoperative tibial segment distalization or thickness of the tibial osteotomy segment as a percentage of total tibial width. Initial gap or resorption gap was not related to the number of screws; all but two patients had two bicortical screw placement confirmed on x‐ray, the remaining two had three bicortical screws.

**Table 1:** Descriptive elements of the four patients with tibial fractures.

Patient	Intraoperative gap mm	Gap resorption mm	% of TTO to total tibial width	Days to fracture	Mechanism of injury	Treatment
1	1.1	3.5	34.2	18	Difficulty with WB Status: Comorbidities of Cerebral palsy and haemophilia	Nonoperative/bracing
2	2.1	3.2	38	84	Fall while on crutches	Open reduction/internal fixation
3	2.4	None	34.2	158	Marching; band practice	Nonoperative/bracing
4	0.5	None	22	201	Testing maximum open chain quad with a dynameter at mid tibia	Nonoperative/bracing

Abbreviations: TTO, tibial tubercle osteotomy; WB, weight bearing.

Six knees had restriction of full knee flexion (arthrofibrosis), which required a surgical intervention of lysis of adhesions. Preop knee flexion ranged from 65°–110° premanipulation. All knees had restoration of functional motion postoperatively (125°−150°), all within 5° of their preoperative knee motion.

## DISCUSSION

The main findings of this study are that operational details of distalization of the tibial tubercle, including length and width of the tibial osteotomized segment, does not negatively contribute to the bony healing of OTB‐ATS interface. It also calls attention to a phenomenon of ‘gap resorption’, as defined as increase in the interface between the distal end of the tibial osteotomy segment, and the tibial shaft after intraoperative screw fixation. Neither the intraoperative gap nor the resorption gap resulted in a measurable effect on bony complications at this site.

The collected demographic variables (tobacco use status, age, BMI and sex), in our study were not found to have any impact on bone healing or complications though the smoking group was too small to make meaningful comparisons. Age showed statistical significance (*p* = 0.02), albeit poor correlation (*r* = 0.29) with days to documented healing. The dimensions of the osteotomized segment (length and thickness %) in this single surgeon's cohort were not found to impact healing or postoperative complication.

Although tibial tubercle osteotomies have proven success in the correction of the patella alta, it is not without associated operative risk. Complications are an ever‐present concern for both patients and surgeons. Our study did not identify a relationship between the technical aspects of the dTTO and specific patient factors, to the occurrence of postoperative complications or cases of delayed union. Distal TTO has been well documented in the literature pertaining to complications [7, 9, 14, 18, 19]. Several studies in the literature have reported an increased risk of complications when a concomitant distalization is performed with a tibial tubercle osteotomy [7, 8, 9, 18]. Payne, et al.'s systematic review of 19 articles [19], evaluated the occurrence of complications across several types of tibial tubercule osteotomies. The authors found an increased complication risk following procedures involving complete detachment of the tibial segment (no hinge) (*N* = 122), which included distalization. This systematic review recorded three occurrences of fractures [19], which is similar to our postoperative tibial fracture incidence of 4% (4/101 knees) diagnosed during postoperative outpatient follow‐up. In a comparative study evaluating tibial tubercle osteotomy with and without distalization, delayed time to union was more frequently encountered with distalization [7].

Current literature is devoid of studies evaluating the distal bony interface in relation to potential complications following dTTO. Specifically, there has been little focus on the effect of the ‘gap’ size, defined as the space between distal end of the osteotomized segment and the anterior tibial cortex (OTB‐ATS) and its relation to healing or complications. The gap was measured intraoperatively at time zero and at post op visits. If there was an increase in the ‘gap’, this was referred to as the ‘resorption’ gap. There was no statistically significant association between gap formation or gap size with the occurrence of postoperative complications.

Specific technical aspects of orthopaedic surgical interventions have been proven to influence healing, functionality and patient clinical outcomes [4]. Our study sought to determine if quantifiable measurements relating to the osteotomy contribute to postoperative complications. The thickness of the osteotomized segment of tibia in relationship to the total width of the proximal tibia was not found to impact the occurrence of fractures or impart a detectable effect on bone healing in our study.

Distalization of tibial tubercle segment did not correlate with any measurable change in rate of healing or incidence of postoperative bony complications in this cohort.

This study was not without limitations. Imaging protocols were used for follow‐up knee radiographs; however, they were not rigorously standardised. Variability exists without a marker, and potentially across technicians. This limits the precision of radiographic measurements. The gap measurement of >2 mm was empirically derived. Despite a standard follow‐up timeline, clinical variation in patient follow‐up occurs, therefore, bone healing data is not specific to the exact time of bone healing, but rather a date by which bone healing occurred. Standard clinical care is 6–8 weeks, but is variable and rarely detailed in studies. All procedures in this study were performed at a single institution and by a single surgeon and may not be reflective of the outcomes of other surgeons performing this same procedure. The smoking population was not adequately powered to allow for meaningful statistical comparison, but the influence of smoking to bone healing is well documented in the literature [13]. Specific attention to the ‘gap’, both intraop at time zero and any increase in the gap postoperatively did not correlate with post op complications in this study. However, with larger numbers of knees and of varying gap sizes, might influence this conclusion.

This study sought to answer questions about the operational aspects of dTTO as it relates to postoperative complications and bone healing. A previous study by our institution showed that the length of distal translation of the tibial tubercle segment up to 15 mm is not associated with postoperative complication, except for an increased incidence arthrofibrosis with translation distance >10 mm [17]. The current study built on this initial study evaluating pertinent clinical question including the average healing time of a tibial tubercle osteotomy and whether the thickness of the tibial segment plays a role in the outcome. Focus was directed to the intraoperative gap size and the resorption gap size, and its (potential) impact on postoperative complications, specifically tibial fracture and time to heal.

The strength of this study is that it was performed by a single surgeon with a consistent clinical and postoperative protocol. Based on information from this study, future evaluations would benefit from measurement of the gap both intra and postoperatively, with a standardised radiographic marker. Regarding the postoperative course, more granular detail on weight‐bearing status and patient compliance would help to inform best practice for postoperative rehabilitation.

## CONCLUSION

The main findings of this study are that operational details of distalization of the tibial tubercle, including length and width of the tibial osteotomized segment or gap measurement both intraop and postop does not negatively contribute to the bony healing of OTB‐ATS interface in this single surgeon study. Demographic variables collected (age, sex, BMI, smoking) did not significantly affect bone healing, though the smoking population was not adequately powered to allow for meaningful statistical comparison. Age was poorly correlated (*r* = 0.29) with days to healing, but statistically significant (*p* = 0.02). All the younger (<18) subjects healed quicker than the older subjects.

## AUTHOR CONTRIBUTIONS

**Arthur Only**: Writing; writing—review and editing. **Marta Engelking**: Data Curation; writing. **Julie Agel**: Data curation; formal analysis; methodology; supervision; validation. **Elizabeth Arendt**: Conceptualisation; data curation; methodology; writing; writing—review and editing; supervision; validation.

## FUNDING INFORMATION

The authors have no funding to report.

## CONFLICT OF INTEREST STATEMENT

The authors declare no conflicts of interest.

## ETHICS STATEMENT

Please include the name of the institutional review board (IRB) and the approval number. If not applicable, please state so. University of Minnesota IRB 0307M50363.

## Data Availability

The data that support the findings of this study are available on request from the corresponding author. The data are not publicly available due to privacy or ethical restrictions.
